# Development of Novel Ecto-Nucleotide Pyrophosphatase/Phosphodiesterase 1 (ENPP1) Inhibitors for Tumor Immunotherapy

**DOI:** 10.3390/ijms23137104

**Published:** 2022-06-26

**Authors:** Xiang Wang, Xing Lu, Daojing Yan, Yajun Zhou, Xiangshi Tan

**Affiliations:** Department of Chemistry, Fudan University, 2005 Songhu Road, Shanghai 200433, China; 17110220109@fudan.edu.cn (X.W.); 17110220108@fudan.edu.cn (X.L.); 16110220094@fudan.edu.cn (D.Y.); 15110220061@fudan.edu.cn (Y.Z.)

**Keywords:** STING, 2′,3′-cGAMP, ENPP1, inhibitor, metastatic breast cancer, tumor immunotherapy

## Abstract

The cyclic guanosine monophosphate–adenosine monophosphate synthase–stimulator of interferon genes–TANK-binding kinase 1–interferon regulating factor 3 (cGAS-STING-TBK1-IRF3) axis is now acknowledged as the major signaling pathway in innate immune responses. However, 2′,3′-cGAMP as a STING stimulator is easily recognized and degraded by ecto-nucleotide pyrophosphatase/phosphodiesterase 1 (ENPP1), which reduces the effect of tumor immunotherapy and promotes metastatic progression. In this investigation, the structure-based virtual screening strategy was adopted to discover eight candidate compounds containing zinc-binding quinazolin-4(3H)-one scaffold as ENPP1 inhibitors. Subsequently, these novel inhibitors targeting ENPP1 were synthesized and characterized by NMR and high-resolution mass spectra (HRMS). In bioassays, 7-fluoro-2-(((5-methoxy-1H-imidazo[4,5-b]pyridin-2-yl)thio)methyl)quina-zolin-4(3H)-one(compound 4**e**) showed excellent activity against the ENPP1 at the molecular and cellular levels, with IC_50_ values of 0.188 μM and 0.732 μM, respectively. Additionally, compound **4e** had superior selectivity towards metastatic breast cancer cells (4T1) than towards normal cells (LO2 and 293T) in comparison with cisplatin, indicating that compound **4e** can potentially be used in metastatic breast cancer therapy. On the other hand, compound **4e** upgraded the expression levels of IFN-β in vivo by preventing the ENPP1 from hydrolyzing the cGAMP to stimulate a more potent innate immune response. Therefore, this compound might be applied to boost antitumor immunity for cancer immunotherapy. Overall, our work provides a strategy for the development of a promising drug candidate targeting ENPP1 for tumor immunotherapy.

## 1. Introduction

**Figure 1:**
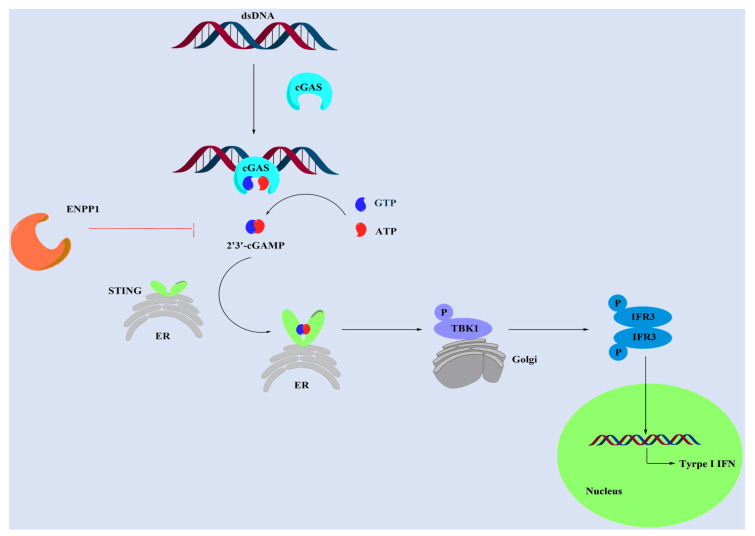
The cGAS-STING-TBK1-IFR3 signaling pathway.

Immune checkpoint inhibitors are an important branch of tumor immunotherapy. They activate adaptive regulatory T cells to kill tumor cells by disrupting immunosuppression signals [1,2]. However, immune checkpoint inhibitors have a few limitations and drug resistance, and their response to noninflammatory cancers is very weak [3,4]. The cGAS-STING-TBK1-IRF3 signaling pathway (Figure 1), a significant part of the innate immune system, is thought to be a broader immunotherapy strategy because it enhances tumor immunogenicity [5,6,7,8]. When the dsDNA of tumor cells enters normal cells, it can be promptly sensed and detected by cyclic guanosine monophosphate (GMP)-adenosine monophosphate (AMP) synthase (cGAS) [9,10]. The subsequent binding of dsDNA with cGAS leads to the activation of cGAS and initiates the catalytic synthesis of 2′,3′-cyclic GMP-AMP (2′,3′-cGAMP) from guanosine triphosphate (GTP) and adenosine triphosphate (ATP) [11,12]. The cGAS-synthesized 2′,3′-cGAMP activates the interferon-gene stimulating factor (STING) in ER to issue in a downstream signaling cascade via the recruitment of TANK-binding kinase 1 (TBK1) and the phosphorylation of the interferon regulatory transcription factor 3 (IRF3) [13,14]. The incitement of IRF3 triggers the production of type I IFN and many other pro-inflammatory cytokines, which can activate CD8^+^ T cells to kill tumor cells [15]. However, 2′,3′-cGAMP, as a STING stimulator, is easily recognized and degraded by ecto-nucleotide pyrophos-phatase/phosphodiesterase 1 (ENPP1), which weakens the effect of tumor immunotherapy [16,17,18].

ENPP1 is involved in different biological processes and hydrolyzes a wide range of phosphodiester bonds, such as nucleoside triphosphates, dinucleotides, cyclic (di-)nucleotides, and nucleotide sugars [19,20]. Li et al. found that ENPP1 hydrolyzed cGAMP (cyclic (di-)nucleotides) and negatively regulated the anti-cancer immune response [17,18]. Moreover, they reported a series of potent ENPP1 inhibitors that could delay tumor growth in a metastatic breast cancer mouse model due to increasing endogenous cGAMP [16]. Furthermore, Bakhoum et al. reported that ENPP1 could hydrolyze extracellular cGAMP to generate AMP and GMP in various metastatic cancers, especially in triple-negative breast cancer (MDA-MB-231 cell line) [21]. The produced AMP was subsequently hydrolyzed into adenosine by CD73 to facilitate cancer-cell migration and extend valid immune suppressive effects [22]. Extracellular cGAMP hydrolysis by ENPP1 transformed an immune stimulatory pathway into an immune-suppressive mechanism that promoted tumor progression. Therefore, finding highly effective ENPP1 inhibitors is a potential therapeutic in metastatic breast cancer.

**Figure 2:**
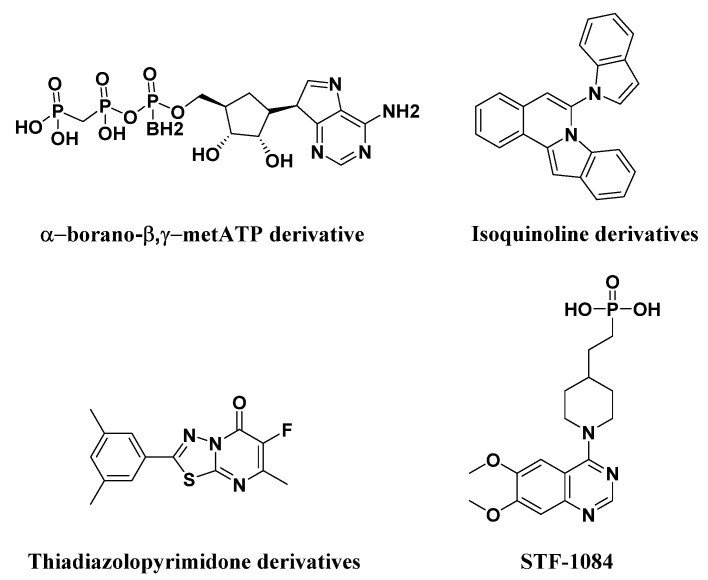
Structures of known ENPP1 inhibitors.

A few nucleotide-derived ENPP1 inhibitors have been reported to date (Figure 2). Sevigny et al. found α-borano-β,γ-metATP derivative, which showed high inhibitory activity against ENPP1 for the replacement of one oxygen atom in the α-phosphate by a borano atom [23]. However, nucleotide-based inhibitors had limited applicability due to their high acidity, resulting in poor peroral bioavailability. As a result, non-nucleotide-based inhibitors might be expected to be ideal lead structures for the development of ENPP1 inhibitors as drugs. Langer et al. concluded that isoquinoline derivatives showed inhibitory activity against ENPP1, whose best inhibitor showed an IC_50_ value of 360 nM [24]. Meanwhile, they also reported a series of thiadiazolopyrimidone derivatives as ENPP1 inhibitors, the best inhibitor displaying an IC_50_ value of 310 nM [25]. Li et al. reported that quinazoline derivatives showed the most potent ENPP1 inhibitors. Among these inhibitors, STF-1084 displayed a K_i_ value of 33 nM against ENPP1, which delays tumor growth in cancer models owing to an increase in the level of endogenous cGAMP [16,17].

As a key component in the early stage of drug discovery, structure-based virtual screening comprised different in silico methodologies to filter a chemical compound library using the three-dimensional structure of the molecular target receptor, which could provide potential candidates for further experimental analysis [26]. CDOCKER is an implementation of a CHARMm-based docking tool using a rigid receptor [27,28], which has attracted increased attention to structure-based virtual screening owing to the advantages of high-precision docking. In this work, we report the discovery of eight ENPP1 inhibitors containing the quinazolin-4(3H)-one scaffold using a structure-based virtual screening strategy, which was carried out by using the CDOCKER protocol with Discovery Studio 2019. However, quinazolin-4(3H)-one is a common heterocyclic alkaloid ring system in medicinal chemistry and was found to possess prominent activity against many cancer cell lines, especially breast cancer and ovarian carcinoma [29,30]. Additionally, quinazolin-4(3H)-one derivatives have been reported to coordinate the catalytic zinc in the active site of metalloenzyme, which was thought to affect the biological role of enzymes [31,32]. Therefore, a series of novel quinazolin-4(3H)-one derivatives were synthesized and characterized by NMR and HRMS. Among these targeted compounds, the most active compound 7-fluoro-2-(((5-methoxy-1H-imidazo[4,5-b]pyri-dine-2-yl)thio)methyl)quinazolin-4(3H)-one(4e) exhibited excellent inhibitory activity at the molecular and cellular levels, with IC_50_ values of 0.188 μM and 0.732 μM against the ENPP1, respectively. Furthermore, compound **4e** was investigated in cytotoxicity studies and pharmacological research in vivo, indicating that compound **4e** can be used as a novel drug candidate targeting ENPP1 for tumor immunotherapy.

## 2. Results and Discussion

### 2.1. Structure-Based Virtual Screening of Novel Hit ENPP1 Inhibitors

**Figure 3:**
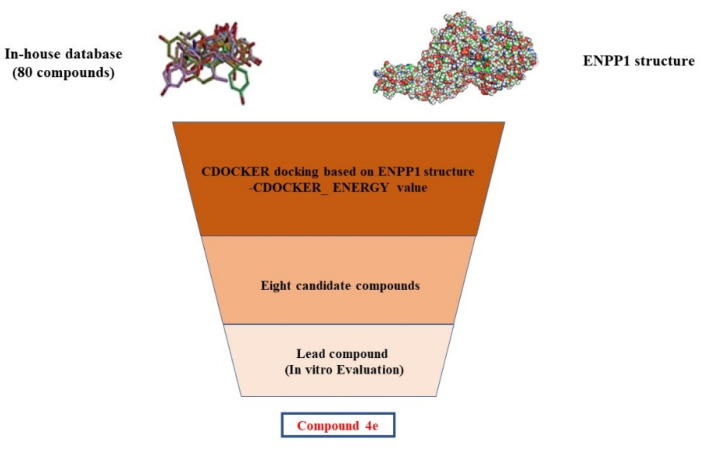
Schematic diagram of the underlying workflow of the ENPP1 inhibitor discovery.

**Figure 4:**
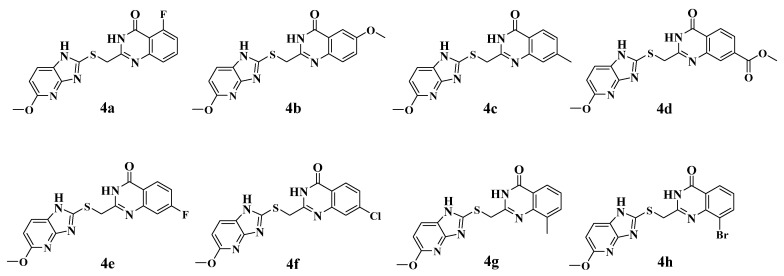
The chemical structures of eight candidate compounds.

The virtual screening process is schematically shown in Figure 3. Virtual screening was performed using the CDOCKER module of Discovery Studio 2019 [33]. The chemical databases comprised the in-house database (80 compounds). The crystal structure of the ENPP1 in complex with AMP (PDB entry 6WFJ) was used for the docking studies, and the ligand (AMP) binding position was utilized as the active site for docking. Next, virtual screening was performed by docking all the prepared ligands at the defined active site using CDOCKER. The RMSD threshold was set to 0.5 Å to ensure that the docking conformation was as diverse as possible [34]. Based on the -CDOCKER_ ENERGY values (Table 1), all the docked poses were ranked and grouped. The top eight compounds were selected from the ranked compounds by the values (the structure of candidate compounds is shown in Figure 4). Clearly, compound **4d** obtained the highest -CDOCKER_ENERGY values (39.904 kcal/mol), indicating that it formed strong-affinity interactions with the ENPP1. However, we elected to synthesize these candidate compounds to carry out biochemical assays.

### 2.2. Synthesis of the Candidate Compounds

**Scheme 1:**
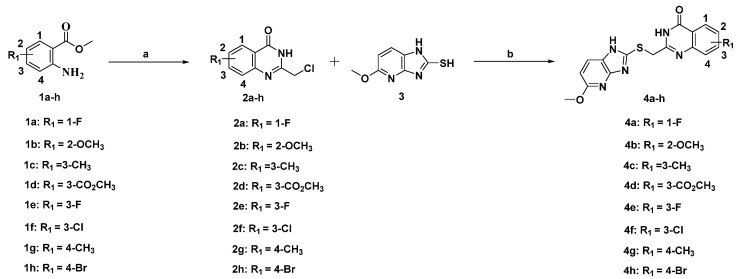
Preparation of compound **4a**–**h**. **Reagents and conditions:** (**a**) 4 M HCl/1,4-dioxane, 100 °C, overnight; (**b**) NaOH, MeOH, rt, overnight.

Scheme 1 shows the preparation of the eight candidate compounds. Commercially available substituted methyl 2-aminobenzoate **1a**–**h** was reacted with chloroacetonitrile in 4 M HCl dioxane at 100 °C to afford the intermediate compounds **2a**–**h** in moderate yields [35]. Next, commercially available 5-methoxy-1H-imidazo[4,5-b]pyridine-2-thiol (compound **3**) was treated with compound **2a**–**h** and sodium hydroxide in methanol to produce the final quinazolin-4(3H)-one derivatives **4a**–**h** in acceptable yields [36].

### 2.3. Inhibition of ENPP1 Enzyme Activity by Compound **4a**–**h**

**Figure 5:**
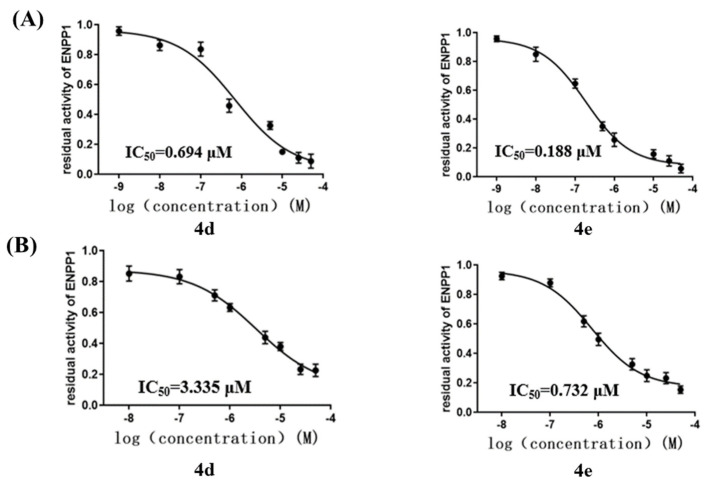
(**A**) IC_50_ values of inhibitors of ENPP1, presented as mean ± SD with three independent enzymatic assays. (**B**) IC_50_ values of inhibitors of ENPP1 in MDA−MB−231 cell line, presented as mean ± SD with three independent cell assays.

An ENPP1 enzymatic inhibitory activity assay was carried out to evaluate the bioactivity of the candidate compounds. As shown in Table 2, these compounds exhibited an inhibition of ENPP1 activity of over 50% at a concentration of 10 μM, which verified the rationality of the molecular docking results. It was obvious that quinazolin-4(3H)-one derivatives displayed inhibitory potency against ENPP1. Herein, we speculate that the carbonyl O atom of the quinazolin-4(3H)-one group might chelate the catalytic zinc ions in the active site to reduce phosphodiesterase activity [37]. According to the results of the enzymatic inhibition, compounds **4d** and **4e** displayed inhibition of over 90%, with IC_50_ values of 0.694 μM and 0.188 μM, respectively (Figure 5A), suggesting that 7-F and 7-CO_2_CH_3_ were the optimal substitutions on the quinazolin-4(3H)-one in comparison with the other inhibitors. When the 7-F and 7-CO_2_CH_3_ on the quinazolin-4(3H)-one scaffold were replaced by a Cl atom (4f), the ENPP1 activity decreased distinctly due to the absence of hydrogen bonds. Furthermore, compound **4h** bearing 8-Br had slightly decreased ENPP1 activity compared with compound **4g** bearing 8-CH_3_, indicating that steric hindrance was critical for the ENPP1 inhibitory activity. In a word, compounds **4d** and **4e** exhibited considerable potency against ENPP1 in enzymatic assays and were chosen for further study to discover the most potent ENPP1 inhibitor.

### 2.4. Cellular ENPP1 Inhibitory Activity by Compound **4a**–**h**

As mentioned above, the ENPP1 was overexpressed in the MDA-MB-231 cell line (TNBC), which facilitated cancer-cell migration by hydrolyzing extracellular cGAMP [21]. In 2018, Nakagawa et al. also reported that MDA-MB-231 cells highly expressed ENPP1 [38]. To our knowledge, there have been no reports on the cellular inhibitory activity of tENPP1 inhibitors in the literature. Herein, we decided to explore the cellular ENPP1 inhibitory activity of compounds **4d** and **4e** in the MDA-MB-231 cell line. As shown in Figure 5B, compounds **4d** and **4e** displayed IC_50_ values of 3.335 μM and 0.732 μM against the ENPP1 in the MDA-MB-231 cell line, respectively, suggesting that these two compounds can be applied to cancer-cell migration by preventing cGAMP from degrading due to ENPP1. However, the results revealed that compound **4e** had the best inhibition in the MDA-MB-231 cell line, exhibiting IC_50_ values of <1 μM against the ENPP1. To sum up, the analyses above led to the discovery of a potent ENPP1 inhibitor (**4e**) that exhibited higher potency at the molecular and cellular levels.

### 2.5. Binding-Mode Analysis

**Figure 6:**
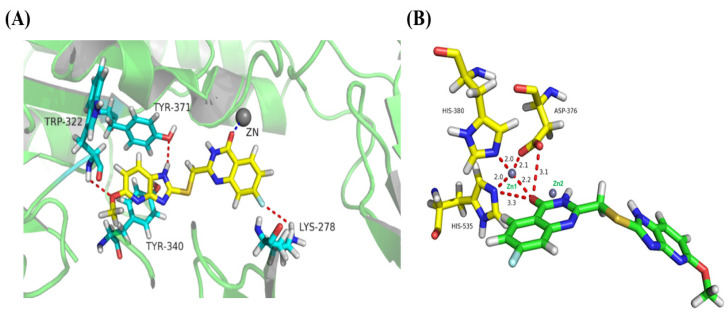
(**A**) Representation of the predicted binding modes of compound **4e** with ENPP1 domain (PDB code 6WFJ). (**B**) OMIT maps of compound **4e** bound to ENPP1. The two Zn atoms are shown as black spheres and Asp376; His380 and His535 are highlighted in stick representation.

The binding mode of compound **4e** within the active site of the ENPP1 predicted by molecular docking is illustrated in Figure 6A. It was noted that compound **4e** was inserted into the catalytic tunnel in a suitable conformation and coordinated with the active-site Zn ion of the ENPP1. The F atom of compound **4e** was predicted to engage in a hydrogen bond with Lys-278, which contributed to the increase in the binding affinity. This might explain why compound **4e** showed better inhibitory activity compared to the other candidate compounds. The results of the molecular modeling also revealed that a hydrogen bond was predicted to exist between the methoxyl of compound **4e** and Trp-322 in the hydrophobic pocket. Another important hydrogen bond was formed between the NH of the imidazole group and Tyr-371. In addition, the binding mode of compound **4e** illustrated π–π stacking interactions between the bicyclic imidazopyridine core and Tyr-340. Altogether, the potency of compound **4e** was driven by its zinc-ion binding, π–π stacking interactions with residues in the hydrophobic pocket, and hydrogen bonds with the active side resides of the ENPP1.

According to the structural information of the ENPP1, two Zn^2+^ ions (Zn1 and Zn2) were tightly bound in the active site among six conserved Asp/His residues (Asp376/His380/His535 and Asp218/Asp423/His424), which played a critical role in the catalytic process [20,39,40]. In 2020, Li et al. reported the structure of ENPP1 in a complex with STF-1084 using X-ray crystallography [16]. They found that STF-1084 occupied the lipid-binding hydrophobic pocket and formed extensive interactions with two zinc ions and six conserved Asp/His residues in the catalytic site. Therefore, we elected to explore the interactions between compound **4e** and the zinc ions in the active site in more detail. As shown in Figure 6B, the carbonyl O atom of compound **4e** was coordinated to the active-site Zn1 ion of ENPP1 with a distance of 2.2 Å and formed H-bond interactions with Asp376 and His535 at distances of 3.1 Å and 3.3 Å, respectively. In conclusion, the quinazolin-4(3H)-one of compound **4e** formed a solid network of interactions with the zinc and Asp376/His380/His535, which was thought to affect the hydrolysis role of the ENPP1. However, the docking results indicated that ENPP1 inhibitors with a zinc-binding quinazolin-4(3H)-one group exhibit good potential.

### 2.6. Cell Viability Assays

Several studies have also reported that the inhibitory activity of the ENPP1 inhibitors could transfer to tumor-cell lines [41,42,43]. As mentioned above, upregulated ENPP1 expression has been associated with metastatic cancer cells, especially in metastatic breast cancer; thus, ENPP1 inhibitors might be useful for the treatment of metastatic breast cancer [16]. However, the overexpression of wild-type ENPP1 could promote cancer migration and metastasis by hydrolyzing the immunotransmitter cGAMP in the 4T1 model [21]. Therefore, breast-cancer-cell (4T1) proliferation assays with compound **4e** were carried out using a standard CCK8 assay. As shown in Table 3, compound **4e** showed the same cytotoxic behavior towards the 4T1 cell line as cisplatin, presenting an IC_50_ value of 2.99 μM. However, compound **4e** exerted a nonsignificant cytotoxic effect on the normal cell lines (LO2 and 293T) at the highest concentration (50 μM) in the experiment, indicating that compound **4e** did not exhibit obvious toxicity. Thus, compound **4e** was considered suitable for anti-tumor research in vivo, especially in the 4T1 breast cancer model. Taking the biological data together, compound **4e** displayed superior selectivity towards 4T1 breast cancer cells than towards normal cells (LO2 and 293T) and might be used in the treatment of metastatic breast cancer.

### 2.7. Molecular Mechanisms of Activity for Compound **4e**

**Figure 7:**
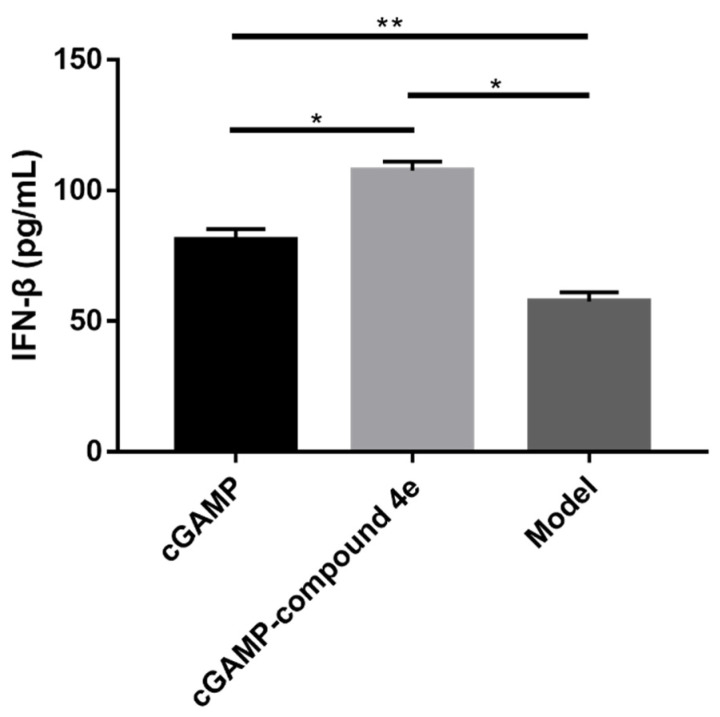
Pharmacodynamic profile of compound **4e** in vivo. IFN-β in serum was measured by ELISA. The values are represented as mean ± SEM, n = 3; (*) *p* < 0.05 and (**) *p* < 0.01.

Li et al. found that ENPP1 inhibitors could increase the half-life of endogenous cGAMP to enhance the natural anti-tumor response. Furthermore, they also reported that the upregulation of IFN-β meant that the cGAS-STING-TBK1-IRF3 signaling pathway was activated to stimulate the innate immune response [17]. In order to explore the pharmacological mechanisms of compound **4e**, we opted to measure the IFN-β from the separated serum by using the ELISA method after the intravenous administration of free cGAMP and cGAMP-compound **4e** in mice, respectively. Compared with the model mice, the upregulation of the IFN-β in the free-cGAMP-treated groups suggested that the cGAMP served as the second message to bind STING and, thus, activated the cGAS-STING-TBK1-IRF3 signaling pathway to stimulate an innate immune response (Figure 7). Additionally, it was found that the expression level of the IFN-β in the cGAMP-compound **4e**-treated group was higher than those in the free-cGAMP-treated group, indicating that compound **4e** could protect cGAMP from degradation to stimulate a more potent innate immune response. As a consequence, compound **4e**, as an ENPP1 inhibitor, can be applied in tumor immunotherapy by preventing cGAMP from hydrolyzing. Altogether, compound **4e** could stimulate a more potent innate immune response while simultaneously reducing the extracellular levels of immuno-suppressive adenosine that promote metastatic progression.

## 3. Materials and Methods

### 3.1. Materials and Reagents

The hENPP1-HIS DNA gene was cloned by Shanghai Generay Biotech Co., Ltd. (Shanghai, China). Cancer cell lines were purchased from the Cell Bank of Chinese Academy of Sciences (Shanghai, China). Thymidine 5′monophosphate p-nitrophenyl ester (pNP-TMP) was purchased from Sigma-Aldrich (Shanghai, China). All commercially obtained reagents and solvents were used directly, without further purification, unless otherwise noted. ^1^H NMR (400 MHz) and ^13^C NMR (101 MHz) spectra were taken on a Bruker AV-400 MHz spectrometer, and chemical shifts were reported in ppm downstream of internal Me_4_Si. High-resolution mass spectra (HRMS) were recorded on a VG ZAB-HS mass spectrometer under electron-spray ionization (ESI). Thin-layer chromatography (TLC) was conducted on aluminum-sheet silica gel Merck 60F254. The spots were visualized using ultraviolet light.

### 3.2. Structure-Based Virtual Screening

In this investigation, the docking method, CDOCKER, was applied on the Discovery Studio 2019 platform. The receptor protein (PDB entry 6WFJ) was prepared by removing the water and some other co-crystallized small molecules, adding hydrogen atoms to the protein, and assigning CHARMm force field in Discovery Studio 2019 software package. After the protein preparation, the binding site of the protein was defined based on volume occupied by the ligand AMP already positioned in the active site. Subsequently, the prepared ligands (according to ligand preparation protocol) from in-house database were docked into the active site of ENPP1. The RMSD threshold was set to 0.5 Å to ensure that the docking conformation was as diverse as possible. Based on the -DOCKER-ENERGY score, all the docked poses were ranked and grouped.

### 3.3. Expression and Purification of Recombinant Human ENPP1

In total, 1 mg hENPP1-HIS DNA using polyethylenimine at a DNA:PEI ratio of 1:3 was transfected into 1 L of 293-F cells in 293 freestyle medium. The medium was harvested after five days and filter-sterilized. Net, the protein was purified on a 10-milliliter Ni affinity column at a flow rate of 10 mL min^−1^, washed with 10 column volumes of buffer 1 (350 mM NaCl with 50 mM Tris pH 8.0) with 20 mM imidazole, and then eluted with 250 mM imidazole in buffer 1. Approximately 1 mg of purified protein was obtained from the 1-liter culture. The quality of the protein purification was validated by SDS-PAGE analysis (see Figure S1) [40].

### 3.4. Chemistry Methods

*2-(Chloromethyl)-5-fluoroquinazolin-4(3H)-one* (**2a**). Anhydrous hydrogen chloride (4 M solution in 1,4-dioxane, 5 mL) was added to a mixture of 2-amino-6-fluorobenzoa-te 1a (0.604 g, 4 mmol, 1eq) and chloroacetonitrile (0.254 mL, 4 mmol, 1eq) and the reaction mixture was stirred at 100 °C overnight. The suspension was cooled to rt and neutralized with saturated NaHCO_3_ solution (20 mL) at ice-bath temperature. The resulting precipitate was washed with water, and dried under vacuum to obtain 0.415 g (49%) of compound **2a** as a brown solid. ^1^H NMR (400 MHz, DMSO) δ 12.62 (s, 1H), 7.81 (td, *J* = 8.0, 5.7 Hz, 1H), 7.49 (d, *J* = 7.8 Hz, 1H), 7.29 (dd, *J* = 7.9, 5.5 Hz, 1H), 4.53 (s, 2H). ^13^C NMR (101 MHz, DMSO) δ 162.25, 159.64, 159.24, 153.87, 150.87, 135.80, 135.70, 123.85, 114.22, 114.01, 43.34.

*2-(Chloromethyl)-6-methoxyquinazolin-4(3H)-one* (**2b**). The desired compound was prepared from methyl 2-amino-5-methoxybenzoate 1b (0.724 g, 4 mmol, 1eq) and chloroacetonitrile (0.254 mL, 4 mmol, 1eq) using the procedure described for compound **2a** in 56% yield as a brown solid. ^1^H NMR (400 MHz, DMSO) δ 12.52 (s, 1H), 7.62 (d, *J* = 8.8 Hz, 1H), 7.49 (s, 1H), 7.42 (d, *J* = 8.7 Hz, 1H), 4.52 (s, 2H), 3.86 (s, 3H). ^13^C NMR (101 MHz, DMSO) δ 161.73, 158.59, 150.34, 143.01, 129.43, 124.38, 122.59, 106.43, 56.08, 43.77.

*2-(Chloromethyl)-7-methylquinazolin-4(3H)-one* (**2c**). The desired compound was prepared from methyl 2-amino-4-methylbenzoate 1c (0.66 g, 4 mmol, 1eq) and chloroacetonitrile (0.254 mL, 4 mmol, 1eq) using the procedure described for compound **2a** in 52% yield as a brown solid. ^1^H NMR (400 MHz, DMSO) δ 12.45 (s, 1H), 7.99 (d, *J* = 8.0 Hz, 1H), 7.46 (s, 1H), 7.35 (d, *J* = 8.0 Hz, 1H), 4.52 (s, 2H), 2.44 (s, 3H). ^13^C NMR (101 MHz, DMSO) δ 161.82, 152.77, 148.78, 145.60, 129.05, 127.35, 126.13, 119.26, 43.71, 21.75.

*Methyl 2-(chloromethyl)-4-oxo-3,4-dihydroquinazoline-7-carboxylate* (**2d**). The desired compound was prepared from dimethyl 2-aminoterephthalate 1d (0.836 g, 4 mmol, 1eq) and chloroacetoneitrile (0.254 mL, 4 mmol, 1eq) using the procedure described for compound **2a** in 43% yield as a brown solid. ^1^H NMR (400 MHz, DMSO) δ 12.80 (s, 1H), 8.23 (d, *J* = 8.2 Hz, 1H), 8.14 (d, *J* = 1.1 Hz, 1H), 8.02 (dd, *J* = 8.2, 1.2 Hz, 1H), 4.56 (s, 2H), 3.91 (s, 3H). ^13^C NMR (101 MHz, DMSO) δ 165.85, 161.50, 154.17, 148.29, 135.51, 128.45, 127.29, 124.83, 53.23, 43.35.

*2-(Chloromethyl)-7-fluoroquinazolin-4(3H)-one* (**2e**). The desired compound was prepared from methyl 2-amino-4-fluorobenzoate 1e (0.676 g, 4 mmol, 1eq) and chloroacetonitrile (0.254 mL, 4 mmol, 1eq) using the procedure described for compound **2a** in 56% yield as a brown solid. ^1^H NMR (400 MHz, DMSO) δ 12.69 (s, 1H), 8.19 (t, *J* = 8.2 Hz, 1H), 7.48 (d, *J* = 8.4 Hz, 1H), 7.41 (t, *J* = 8.7, 1H), 4.53 (s, 2H). ^13^C NMR (101 MHz, DMSO) δ 167.49, 164.99, 161.27, 154.36, 150.99, 129.53, 129.42, 118.76, 116.37, 116.14, 113.08, 112.86, 43.51.

*7-Chloro-2-(chloromethyl)quinazolin-4(3H)-one* (**2f**). The desired compound was prepared from methyl 2-amino-4-chlorobenzoate 1f (0.742 g, 4 mmol, 1eq) and chloroacetonitrile (0.254 mL, 4 mmol, 1eq) using the procedure described for compound **2a** in 55% yield as a brown solid. ^1^H NMR (400 MHz, DMSO) δ 12.73 (s, 1H), 8.10 (d, *J* = 8.5 Hz, 1H), 7.74 (s, 1H), 7.57 (d, *J* = 8.5 Hz, 1H), 4.54 (s, 2H). ^13^C NMR (101 MHz, DMSO) δ 161.34, 154.37, 149.86, 139.68, 128.38, 127.97, 126.90, 120.53, 43.48.

*2-(Chloromethyl)-8-methylquinazolin-4(3H)-one* (**2g**). The desired compound was prepared from methyl 2-amino-3-methylbenzoate 1g (0.66 g, 4 mmol, 1eq) and chloroacetonitrile (0.254 mL, 4 mmol, 1eq) using the procedure described for compound **2a** in 51% yield as a brown solid. ^1^H NMR (400 MHz, DMSO) δ 12.55 (s, 1H), 7.95 (d, *J* = 7.6 Hz, 1H), 7.68 (d, *J* = 7.5 Hz, 1H), 7.41 (t, *J* = 7.6 Hz, 1H), 4.55 (s, 2H), 2.52 (s, 3H). ^13^C NMR (101 MHz, DMSO) δ 162.22, 151.61, 147.09, 135.96, 135.49, 127.16, 123.94, 121.66, 44.00, 17.55.

*8-Bromo-2-(chloromethyl)quinazolin-4(3H)-one* (**2h**). The desired compound was prepared from methyl 2-amino-3-bromobenzoate 1h (0.92 g, 4 mmol, 1eq) and chloroacetonitrile (0.254 mL, 4 mmol, 1eq) using the procedure described for compound **2a** in 51% yield as a brown solid. ^1^H NMR (400 MHz, DMSO) δ 12.83 (s, 1H), 8.15 (d, *J* = 7.7 Hz, 1H), 8.11 (d, *J* = 7.9 Hz, 1H), 7.44 (t, *J* = 7.8 Hz, 1H), 4.56 (s, 2H). ^13^C NMR (101 MHz, DMSO) δ 161.53, 153.80, 146.18, 138.59, 128.56, 126.17, 123.44, 122.34, 43.72.

*5-Fluoro-2-(((5-methoxy-1H-imidazo[4,5-b]pyridin-2-yl)thio)methyl)quinazol-in-4(3H)-one* (**4a**). Compound **2a** (0.212 g, 1 mmol, 1eq) and 5-methoxy-1H-imidazo[4,5-b]pyridine-2-thiol (compound **3**, 0.182 g, 1 mmol, 1eq) were dissolved in MeOH (7 mL) and treated with NaOH (0.2 g, 5 mmol, 5eq). The reaction mixture was stirred at room temperature overnight, and the solvent was removed under reduced pressure. The residue was purified by silica gel column chromatography (dichloromethane− methanol, gradient 100:0 → 100:5) to obtain the desired product 4a (0.179 g, 50% yield). ^1^H NMR (400 MHz, DMSO) δ 13.20 (s, 1H), 12.62 (s, 1H), 7.74–7.65 (m, 2H), 7.39 (d, *J* = 8.1 Hz, 1H), 7.24 (t, *J* = 7.5 Hz, 1H), 6.63 (d, *J* = 8.5 Hz, 1H), 4.48 (s, 2H), 3.86 (s, 3H). ^13^C NMR (101 MHz, DMSO) δ 162.61, 162.18, 159.56, 158.77, 154.74, 148.81, 147.10, 144.62, 136.10, 136.00, 126.03, 122.11, 121.84, 114.23, 114.03, 110.91, 110.84, 109.63, 54.54, 35.44. ESI-MS m/z calcd for C16H12FN5O2S^+^ 358.0768 found 358.0754 [M + H] ^+^.

*6-Methoxy-2-(((5-methoxy-1H-imidazo[4,5-b]pyridin-2-yl)thio)methyl)quinaz-olin-4(3H)-one* (**4b**). The desired compound was prepared from compound **2b** (0.224 g, 1 mmol, 1eq), compound **3** (0.182 g, 1 mmol, 1eq), and NaOH (0.2 g, 5 mmol, 5eq) using the procedure described for compound **4a** in 50% yield as a white solid. ^1^H NMR (400 MHz, CDCl3) δ 7.85 (d, *J* = 8.4 Hz, 1H), 7.66 (s, 1H), 7.64 (d, *J* = 8.9 Hz, 1H), 7.36 (d, *J* = 8.3 Hz, 1H), 6.69 (d, *J* = 8.6 Hz, 1H), 4.42 (s, 2H), 3.98 (s, 3H), 3.93 (s, 3H). ^13^C NMR (101 MHz, DMSO) δ 161.58, 161.15, 158.35, 151.46, 148.72, 148.53, 142.50, 129.36, 128.66, 126.03, 125.88, 124.34, 122.32, 106.55, 56.12, 53.96, 35.20. ESI-MS m/z calcd for C17H15N5O3S^+^ 370.0968 found 370.0973 [M + H]^+^.

*2-(((5-Methoxy-1H-imidazo[4,5-b]pyridin-2-yl)thio)methyl)-7-methylquinazoli-n-4(3H)-one* (**4c**). The desired compound was prepared from compound **2c** (0.208 g, 1 mmol, 1eq), compound **3** (0.182 g, 1 mmol, 1eq), and NaOH (0.2 g, 5 mmol, 5eq) using the procedure described for compound **4a** in 57% yield as a white solid. ^1^H NMR (400 MHz, DMSO) δ 12.48 (s, 1H), 7.96 (d, *J* = 8.0 Hz, 1H), 7.79 (d, *J* = 8.1 Hz, 1H), 7.38 (s, 1H), 7.30 (d, *J* = 8.1 Hz, 1H), 6.61 (d, *J* = 8.5 Hz, 1H), 4.48 (s, 2H), 3.84 (s, 3H), 2.41 (s, 3H). ^13^C NMR (101 MHz, DMSO) δ 162.74, 160.42, 156.38, 147.03, 145.29, 144.57, 142.66, 130.02, 126.79, 126.32, 122.53, 122.06, 118.45, 110.05, 54.54, 34.36, 21.83. ESI-MS m/z calcd for C17H15N5O2S^+^ 354.1019 found 354.1023 [M + H]^+^.

*Methyl 2-(((5-methoxy-1H-imidazo[4,5-b]pyridin-2-yl)thio)methyl)-4-oxo-3,4-dihydroquina-zoline-7-carboxylate* (**4d**). The desired compound was prepared from compound **2d** (0.252 g, 1 mmol, 1eq), compound **3** (0.182 g, 1 mmol, 1eq), and NaOH (0.2 g, 5 mmol, 5eq) using the procedure described for compound **4a** in 36% yield as a white solid. ^1^H NMR (400 MHz, DMSO) δ 13.45 (s, 1H), 12.82 (s, 1H), 8.20 (d, *J* = 8.2 Hz, 1H), 8.03 (s, 1H), 7.96 (d, *J* = 8.2 Hz, 1H), 7.79 (d, *J* = 8.4 Hz, 1H), 6.62 (d, *J* = 8.6 Hz, 1H), 4.53 (s, 2H), 3.90 (s, 3H), 3.85 (s, 3H). ^13^C NMR (101 MHz, DMSO) δ 166.55, 165.62, 162.67, 161.10, 154.71, 147.13, 144.48, 136.80, 135.33, 127.32, 127.07, 126.03, 124.57, 121.75, 109.74, 54.53, 53.19, 35.81. ESI-MS m/z calcd for C18H15N5O4S^+^ 398.0918 found 398.0921 [M + H]^+^.

*7-Fluoro-2-(((5-methoxy-1H-imidazo[4,5-b]pyridin-2-yl)thio)methyl)quinazolin-4(3H)-one* (**4e**). The desired compound was prepared from compound **2e** (0.212 g, 1 mmol, 1eq), compound **3** (0.182 g, 1 mmol, 1eq), and NaOH (0.2 g, 5 mmol, 5eq) using the procedure described for compound **4a** in 68% yield as a white solid. ^1^H NMR (400 MHz, DMSO) δ 13.22 (s, 1H), 12.69 (s, 1H), 8.22–8.10 (m, 1H), 7.83 (s, 1H), 7.48–7.36 (m, 2H), 6.63 (d, *J* = 8.6 Hz, 1H), 4.51 (s, 2H), 3.86 (s, 3H). ^13^C NMR (101 MHz, DMSO) δ 167.35, 164.84, 162.59, 161.10, 154.45, 150.11, 149.98, 147.43, 144.67, 129.54, 129.43, 126.20, 121.88, 118.44, 116.05, 115.82, 112.21, 111.99, 109.60, 54.52, 36.00. ESI-MS m/z calcd for C16H12FN5O2S^+^ 358.0768 found 358.0762 [M + H]^+^.

*7-Chloro-2-(((5-methoxy-1H-imidazo[4,5-b]pyridin-2-yl)thio)methyl)quinazolin-4(3H)-one* (**4f**). The desired compound was prepared from compound **2f** (0.23 g, 1 mmol, 1eq), compound **3** (0.182 g, 1 mmol, 1eq), and NaOH (0.2 g, 5 mmol, 5eq) using the procedure described for compound **4a** in 53% yield as a white solid. ^1^H NMR (400 MHz, DMSO) δ 12.81 (s, 2H), 8.10 (d, *J* = 8.5 Hz, 1H), 7.80 (d, *J* = 8.6 Hz, 1H), 7.64 (s, 1H), 7.54 (d, *J* = 8.5 Hz, 1H), 6.63 (d, *J* = 8.6 Hz, 1H), 4.51 (s, 2H), 3.86 (s, 3H). ^13^C NMR (101 MHz, DMSO) δ 162.65, 161.03, 154.97, 148.40, 147.25, 144.47, 139.70, 128.54, 127.82, 126.03, 125.55, 121.70, 120.30, 109.69, 54.56, 35.87. ESI-MS m/z calcd for C16H12ClN5O2S^+^ 374.0473 found 374.0489 [M + H]^+^.

*2-(((5-Methoxy-1H-imidazo[4,5-b]pyridin-2-yl)thio)methyl)-8-methylquinazoli-n-4(3H)-one* (**4g**). The desired compound was prepared from compound **2g** (0.208 g, 1 mmol, 1eq), compound **3** (0.182 g, 1 mmol, 1eq), and NaOH (0.2 g, 5 mmol, 5eq) using the procedure described for compound **4a** in 52% yield as a white solid. ^1^H NMR (400 MHz, CDCl3) δ 8.16 (d, *J* = 7.8 Hz, 1H), 7.78 (d, *J* = 8.1 Hz, 1H), 7.63 (d, *J* = 7.4 Hz, 1H), 7.39 (t, *J* = 7.6 Hz, 1H), 6.62 (d, *J* = 8.7 Hz, 1H), 4.41 (s, 2H), 3.95 (s, 3H), 2.68 (s, 3H). ^13^C NMR (101 MHz, DMSO) δ 162.66, 161.88, 151.90, 148.03, 146.07, 144.00, 135.45, 134.81, 126.85, 125.79, 123.97, 121.26, 121.14, 109.77, 54.53, 35.79, 16.89. ESI-MS m/z calcd for C17H15N5O2S^+^ 354.1019 found 354.1028 [M + H]^+^.

*8-Bromo-2-(((5-methoxy-1H-imidazo[4,5-b]pyridin-2-yl)thio)methyl)quinazoli-n-4(3H)-one* (**4h**). The desired compound was prepared from compound **2h** (0.274 g, 1 mmol, 1eq), compound **3** (0.182 g, 1 mmol, 1eq), and NaOH (0.2 g, 5 mmol, 5eq) using the procedure described for compound **4a** in 41% yield as a white solid. ^1^H NMR (400 MHz, DMSO) δ 13.22 (s, 1H), 12.85 (s, 1H), 8.08 (d, *J* = 7.7 Hz, 2H), 7.77 (d, *J* = 8.1 Hz, 1H), 7.39 (t, *J* = 7.7 Hz, 1H), 6.61 (d, *J* = 8.5 Hz, 1H), 4.50 (s, 2H), 3.80 (s, 3H). ^13^C NMR (101 MHz, DMSO) δ 162.67, 161.32, 153.70, 147.97, 145.76, 143.92, 138.28, 128.13, 126.14, 125.80, 123.13, 121.52, 121.13, 109.79, 54.55, 35.86. ESI-MS m/z calcd for C16H12BrN5O2S^+^ 419.9948 found 419.9933 [M + H]^+^.

### 3.5. Enzyme-Based ENPP1 Inhibitory Assays

The initial screenings were carried out at 37 °C in a total volume of 0.1 mL of the following incubation mixture: 1 mM CaCl_2_, 200 μM ZnCl_2_, 50 mM Tris, pH 9.0, and 10 μM of each test compound. The enzyme reactions began with the addition of 20 ng of human soluble ENPP1, then incubated at 37 °C for 10 min and initiated by the addition of 200 μM pNP-TMP. The production of p-nitrophenol was measured after 30 min reaction at 405 nm. Blank values were subtracted from these data. For the blank samples, the enzyme was added at the end of the reaction and read immediately. The IC_50_ values of test compounds were calculated by plotting three independent experiments using the program Prism 5.0. Incubation and operation conditions remained the same as those described above [37,44].

### 3.6. Cellular ENPP1 Enzymatic Inhibition Assays

MDA-MB-231 (1.0 × 10^4^ cells/well) were grown on 96-well microplates (IWAKI) overnight in the culture medium (100 μL/well) in a humidified incubator containing 5% CO_2_ in air at 37 °C for 24 h. The plates were washed with D-Hanks (100 μL) twice, and fresh D-Hanks (80 μL) was added. 5% DMSO or test compound (10 μL) was added to each well, and eventually pNP-TMP (2 mM, 10 μL, final 200 μM) was added. After 4 h incubation at 37 °C, the amounts of released p-nitrophenolate were measured at 405 nm. The IC_50_ values of test compounds were calculated by plotting three independent experiments using the program Prism 5.0 [38].

### 3.7. Molecular Docking Study

Docking simulations were performed to study the binding pattern of compound **4e** to ENPP1 pocket. ENPP1 (PDB entry: 6WFJ) were downloaded from Protein Data Bank (PDB). The Docking simulation was performed using by CDOCKER. Hydrogen atoms were added to the proteins by using Discovery Studio 2019 (Accelrys Inc., San Diego, CA, USA). Charmm force field was assigned. The binding site was defined as a sphere containing the residues that remained within 10 Å of the ligand, an area large enough to cover the ligand-binding region at the active site. The docking results showed the optimized molecular docking model with the receptor proteins and gave a score. The image was created using PyMOL.

### 3.8. Cell Culture and Proliferation Assays

In total, 1 × 10^4^ cells (4T1, LO2 and 293T) were plated in 96-well flat-bottom plates in a final volume of 200 μL DMEM supplemented with 10% FBS and cultured for 24 h. Next, the culture was replaced by fresh DMEM medium (200 μL) and the compound (10 μL) was added. After forty-eight hours, the medium was replaced by PBS (180 μL) mixture with CCK8 solution (20 μL) in each well. Cell viability was evaluated by testing the OD_450_ nm. The data were processed by GraphPad Prism 7.0 [45,46].

### 3.9. Pharmacological Research on ENPP1 Inhibitors in Mice

The animal studies were conducted with the approval of the Animal Care and Use Committee at Fudan University. BALB/c nude mice (6−8 weeks) were randomized into three groups (n = 3, with a total tumor number of 9). Free cGAMP (at dose of 5 mg/kg) and cGAMP-compound **4e** (at doses of 5 mg/kg cGAMP and 0.5 mg/kg compound **4e**), respectively, were injected intravenously (i.v.) into mice via tail vein. After i.v. administration, mouse blood samples were collected from ophthalmic vein after 0.5 h and centrifuged at 500× *g* for 20 min. The concentration of IFN-β in the serum was determined by mouse ELISA kits, according to the instruction manual [47].

## 4. Conclusions

In this study, the structure-based docking strategy was used to screen for novel ENPP1 inhibitors. According to the docking results, eight candidate compounds containing the quinazolin-4(3H)-one scaffold were selected to carry out the bioassays. The recombinant ENPP1 was expressed and purified to evaluate the enzymatic inhibitory activity of the compounds. Of these compounds, compound **4d** and compound **4e** exhibited excellent inhibitory activity against the ENPP1, with IC_50_ values of 0.694 μM and 0.188 μM, respectively. Additionally, two inhibitors also displayed remarkable inhibitory activity against the ENPP1 in the MDA-MB-231 cell line. However, compound **4e** showed excellent inhibitory activity against the ENPP1 at the molecular and cellular levels, exhibiting IC_50_ values of <1 μM. Through binding-mode analysis, compound **4e** was predicted to form a coordination bond, a hydrogen bond, and hydrophobic interaction with the ENPP1, accounting for obvious enzymatic inhibitory activity at the molecular and cellular levels. On the other hand, compound **4e** showed high cytotoxic activity against the 4T1 cell line and negligible cytotoxic effects against the normal cell lines (LO2 and 293T), indicating that compound **4e** can potentially be employed in metastatic breast cancer therapy. Finally, we found that since compound **4e** could enhance the expression levels of IFN-β in vivo by preventing cGAMP from degrading due to ENPP1, it might be applied in tumor immunotherapy.

The inhibition of ENPP1 differs from the pharmacologic activation of STING in a few crucial ways, even though they all elicit anti-tumor immunity through the cGAS-STING-TBK1-IRF3 signaling pathway. On one hand, ENPP1 inhibitors are capable of maintaining cellular endogenous cGAMP levels to inhibit cancer migration and metastasis. On the other hand, ENPP1 inhibitors have a larger therapeutic window, since the systemic administration of STING agonists may cause potential side effects. In summary, compound **4e**, as a novel drug candidate targeting ENPP1, could regulate endogenous cGAMP and, thus, deserves further anti-tumor research in vivo. In addition, the synergistic effects of the combination of ENPP1 inhibitors and the STING agonist, cGAMP, should be studied for its therapeutic potential in cancer.

## Figures and Tables

**Table 1:** The -CDOCKER_ ENERGY values of eight candidate compounds.

Cpd.	-CDOCKER_ ENERGY	Cpd.	-CDOCKER_ ENERGY
**4a**	33.483	**4b**	34.596
**4c**	35.391	**4d**	39.904
**4e**	34.405	**4f**	33.617
**4g**	33.616	**4h**	33.293

**Table 2:** Enzymatic inhibition of ENPP1 at 10 μM **4a**–**h**.

Cpd.	Inhibition (%) @ 10 μM	Cpd.	Inhibition (%) @ 10 μM
**4a**	72.2	**4b**	76.5
**4c**	75.4	**4d**	92.6
**4e**	90.5	**4f**	52.9
**4g**	72.7	**4h**	55.5

The data are presented as the mean ± SD with three independent assays.

**Table 3:** Determination of IC_50_ (μM) values for compound **4e** and cisplatin against 4T1 cell line; cytotoxic effect of compound **4e** and cisplatin (% inhibition) against LO2 and 293T cell lines at 50 μM.

Codes	4T1	LO2	293T
Compound **4e**	2.99 μM	15.33%	9.35%
**Cisplatin**	3.2 μM	57%	52%

The data are presented as the mean ± SD with three independent assays.

## Data Availability

The data presented in this study are available upon request from the corresponding authors.

## Supplementary Materials

The following supporting information can be downloaded at: https://www.mdpi.com/article/10.3390/ijms23137104/s1.
