# Supplementary material for: Development of Novel Ecto-Nucleotide Pyrophosphatase/Phosphodiesterase 1 (ENPP1) Inhibitors for Tumor Immunotherapy
Source: Int J Mol Sci. 2022 Jun 26;23(13):7104. doi: 10.3390/ijms23137104 (PMC9266353; doi:10.3390/ijms23137104)
Supplement: Supplementary file 1 [file ijms-23-07104-s001.zip › ijms-1766322-supplementary.pdf]

Development of Novel Ecto-Nucleotide Pyrophosphatase/ Phosphodiesterase 1 (ENPP1) Inhibitors for Tumor Immunotherapy

Xiang Wang, Xing Lu, Daojing Yan , Yajun Zhou and Xiangshi Tan*
Department of Chemistry, Fudan University, Shanghai 200433, China

* Corresponding authors.
E-mail addresses: xstan@fudan.edu.cn

Table of contents

Fig S1 2
NMR spectrum and HMRS spectrum. 3
Gene sequence. 11
Peptide sequence 13

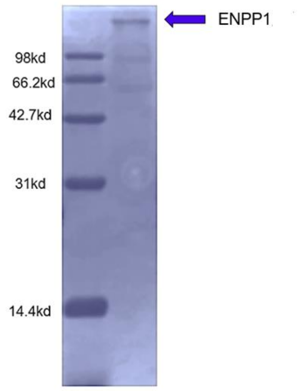

Fig. S1 The quality of protein purification was validated by SDS–PAGE analysis

NMR spectrum and HMRS spectrum

4a

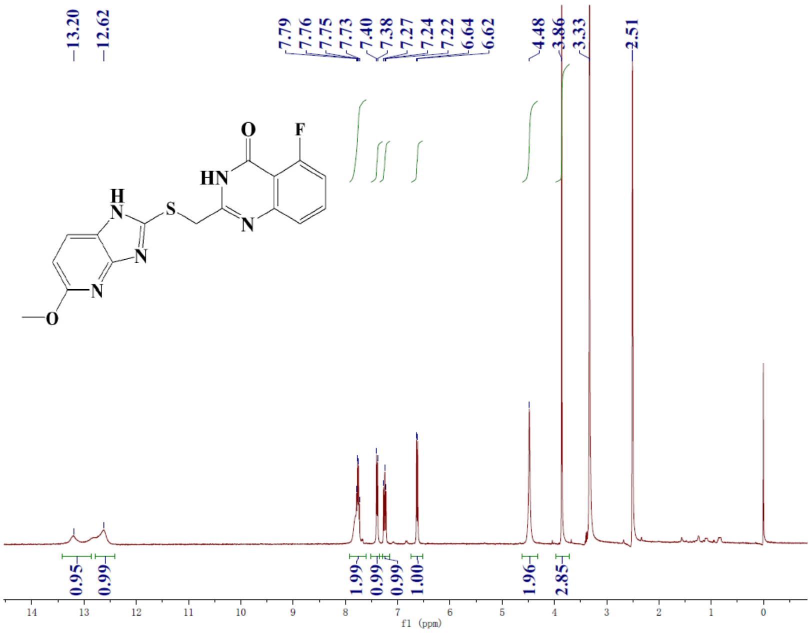

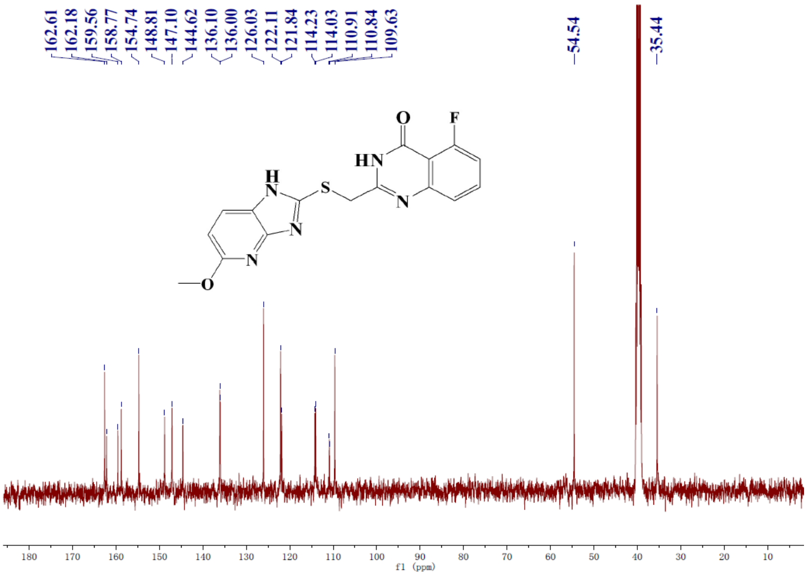

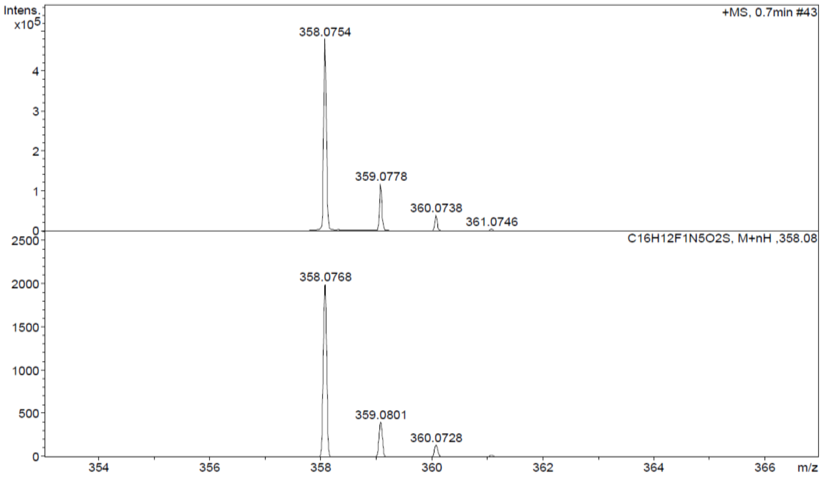

4b

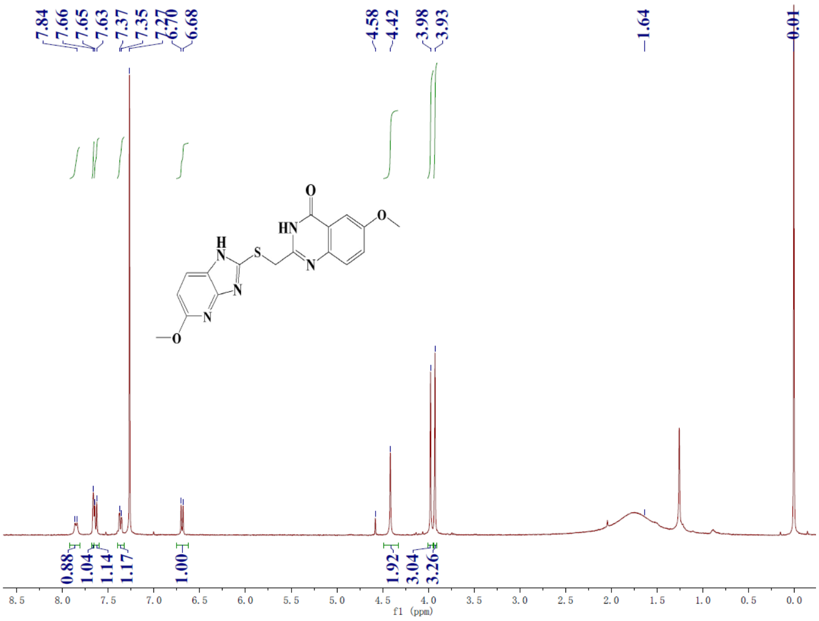

7.84 7.66 7.65 7.63 7.37 7.35 7.27 6.70 6.68
4.58 4.42 3.98 3.93
1.64
0.01
0.88 1.04 1.14 1.17 1.00 1.92 3.04 3.26
8.5 8.0 7.5 7.0 6.5 6.0 5.5 5.0 4.5 4.0 3.5 3.0 2.5 2.0 1.5 1.0 0.5 0.0
f1 (ppm)

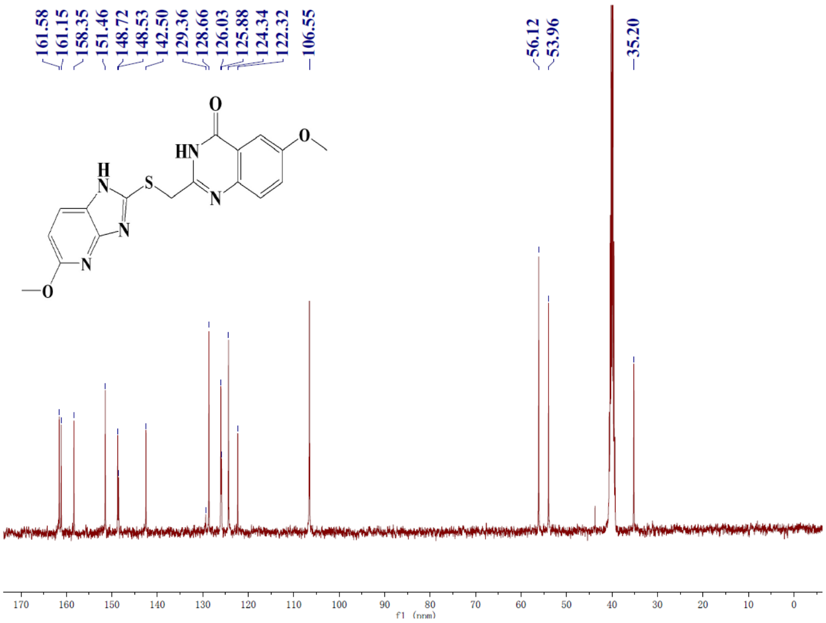

161.58 161.15 158.35 151.46 148.72 148.53 142.50 129.36 128.66 126.03 125.88 124.34 122.32 106.55
56.12 53.96
35.20
170 160 150 140 130 120 110 100 90 80 70 60 50 40 30 20 10 0
f1 (ppm)

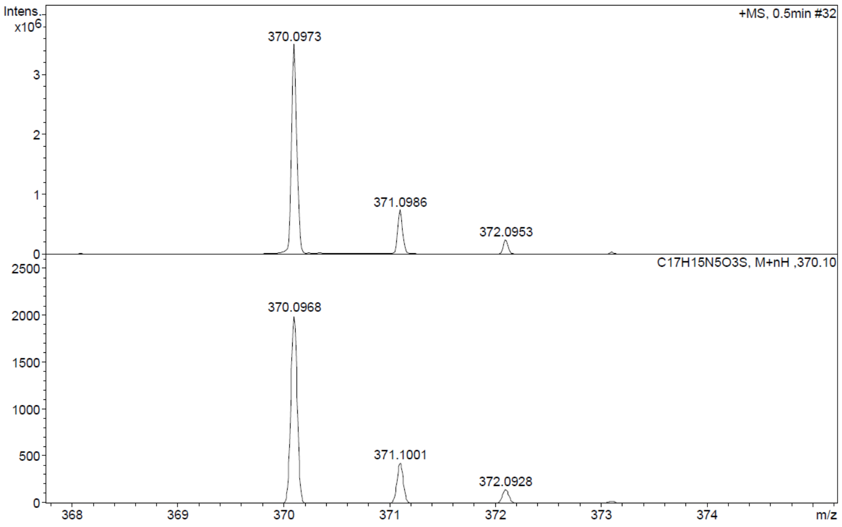

Intens. x10^6
+MS, 0.5min #32
370.0973
371.0986
372.0953
C17H15N5O3S, M+nH ,370.10
370.0968
371.1001
372.0928
368 369 370 371 372 373 374 m/z

4c

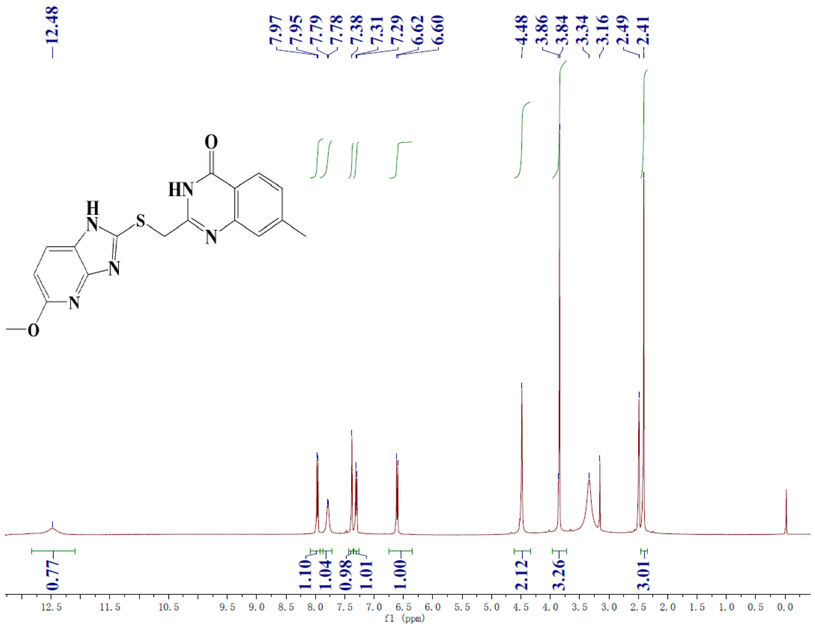
12.48
7.97
7.95
7.79
7.78
7.38
7.31
7.29
6.62
6.60
4.48
3.86
3.84
3.34
3.16
2.49
2.41
HN
O
H
N
S
N
N
N
O
0.77
1.10
1.04
0.98
1.01
1.00
2.12
3.26
3.01
f1 (ppm)

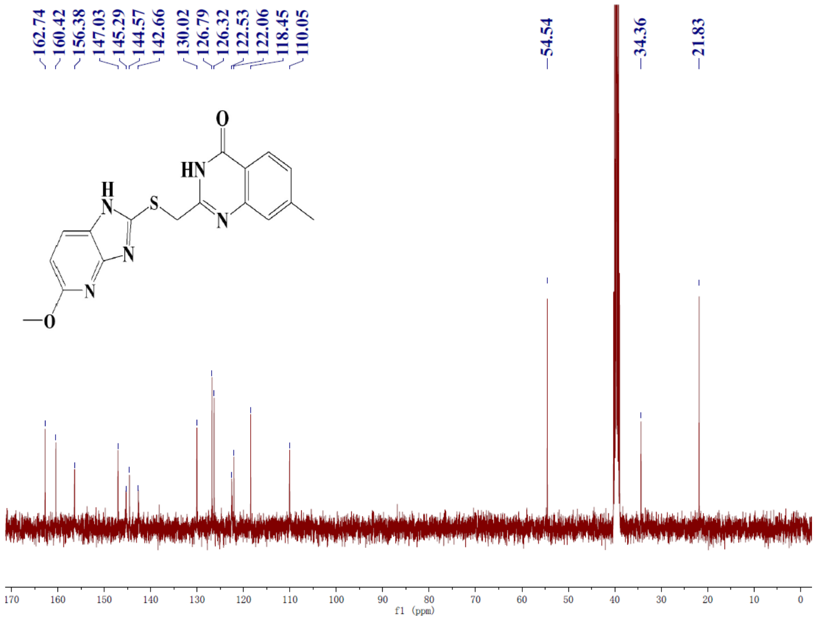
162.74
160.42
156.38
147.03
145.29
144.57
142.66
130.02
126.79
126.32
122.53
122.06
118.45
110.05
54.54
34.36
21.83
HN
O
H
N
S
N
N
N
O
f1 (ppm)

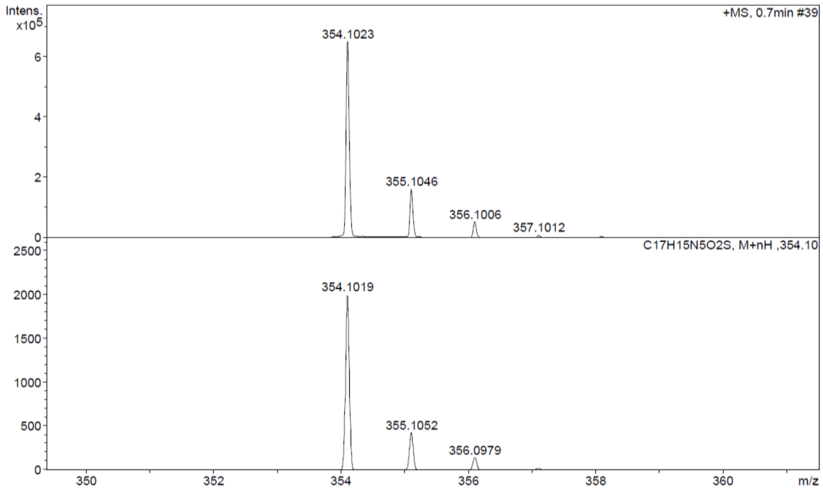
Intens.
x10^5
+MS, 0.7min #39
354.1023
355.1046
356.1006
357.1012
C17H15N5O2S, M+nH ,354.10
354.1019
355.1052
356.0979
m/z

4d

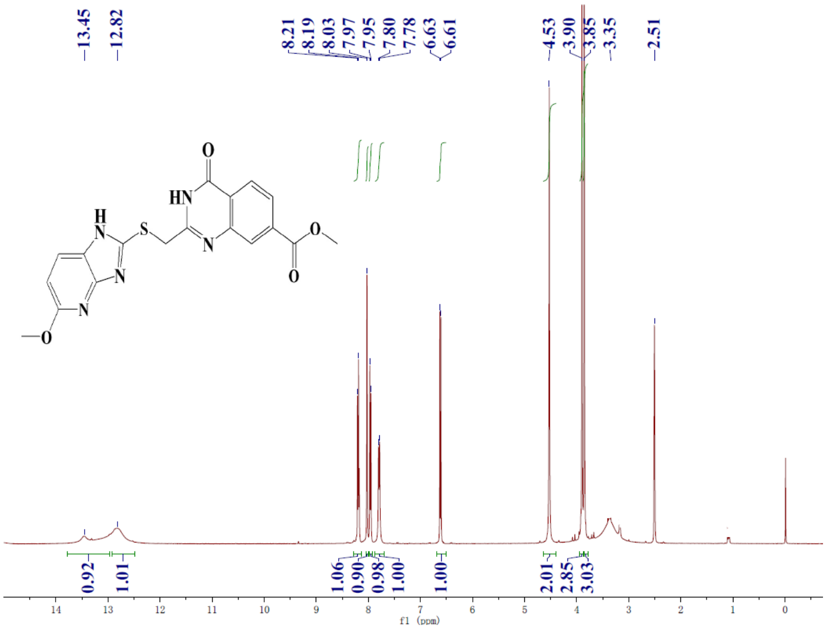
13.45
12.82
8.21
8.19
8.03
7.97
7.95
7.80
7.78
6.63
6.61
4.53
3.90
3.85
3.35
2.51
HN
O
N
S
H
N
N
N
O
O
O
0.92
1.01
1.06
0.90
0.98
1.00
1.00
2.01
2.85
3.03
14
13
12
11
10
9
8
7
6
5
4
3
2
1
0
f1 (ppm)

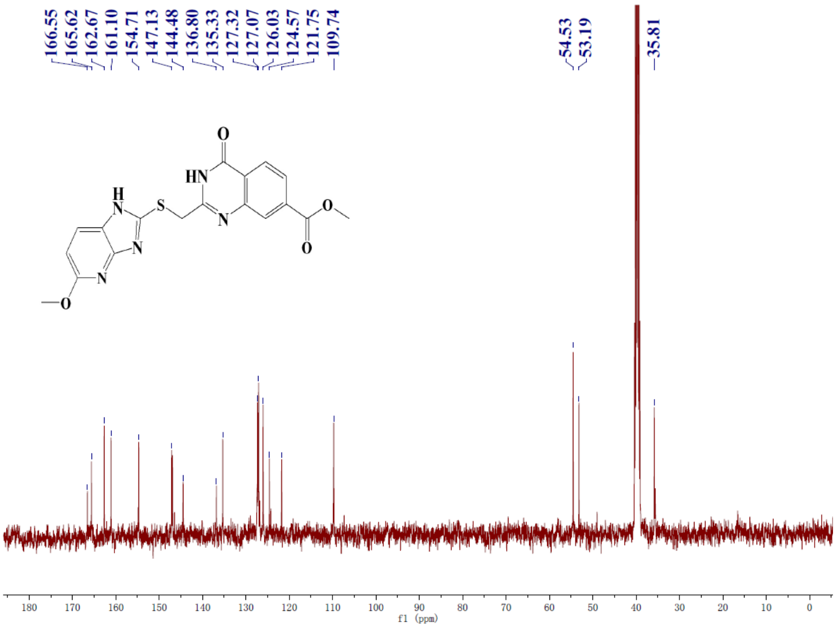
166.55
165.62
162.67
161.10
154.71
147.13
144.48
136.80
135.33
127.32
127.07
126.03
124.57
121.75
109.74
54.53
53.19
35.81
180
170
160
150
140
130
120
110
100
90
80
70
60
50
40
30
20
10
0
f1 (ppm)

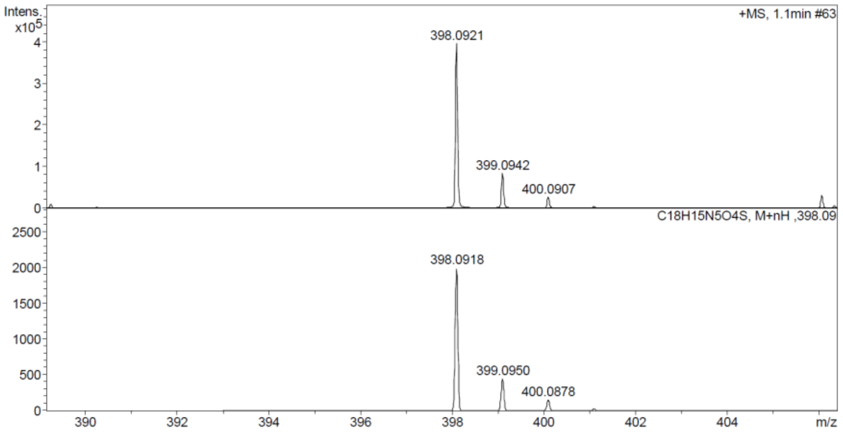
Intens.
x10⁵
+MS, 1.1min #63
398.0921
399.0942
400.0907
C18H15N5O4S, M+nH ,398.09
398.0918
399.0950
400.0878
390
392
394
396
398
400
402
404
m/z

4e

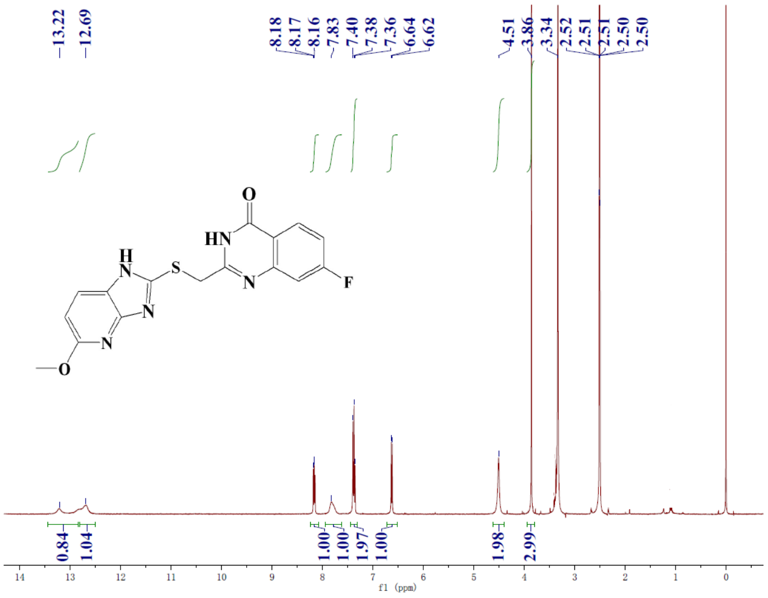
13.22
12.69
8.18
8.17
8.16
7.83
7.40
7.38
7.36
6.64
6.62
4.51
3.86
3.34
2.52
2.51
2.51
2.50
2.50
0.84
1.04
1.00
1.00
1.97
1.00
1.98
2.99
f1 (ppm)

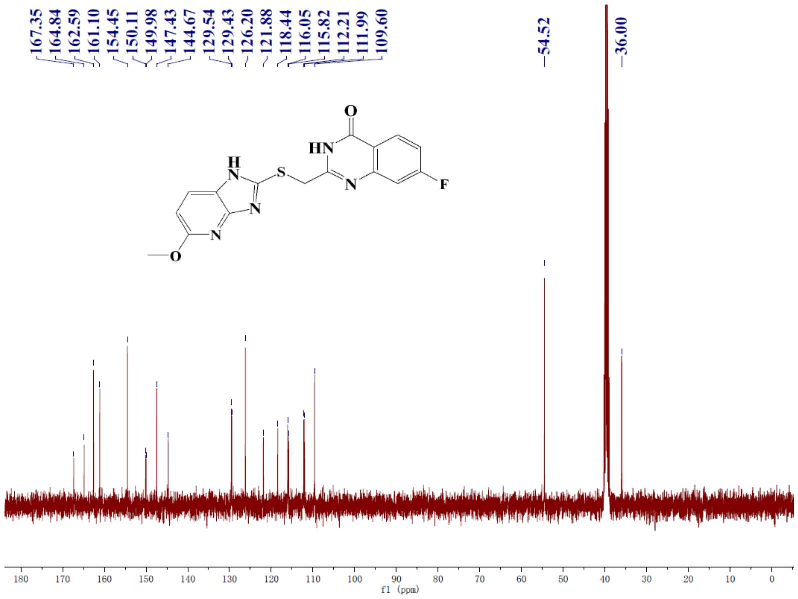
167.35
164.84
162.59
161.10
154.45
150.11
149.98
147.43
144.67
129.54
129.43
126.20
121.88
118.44
116.05
115.82
112.21
111.99
109.60
54.52
36.00
f1 (ppm)

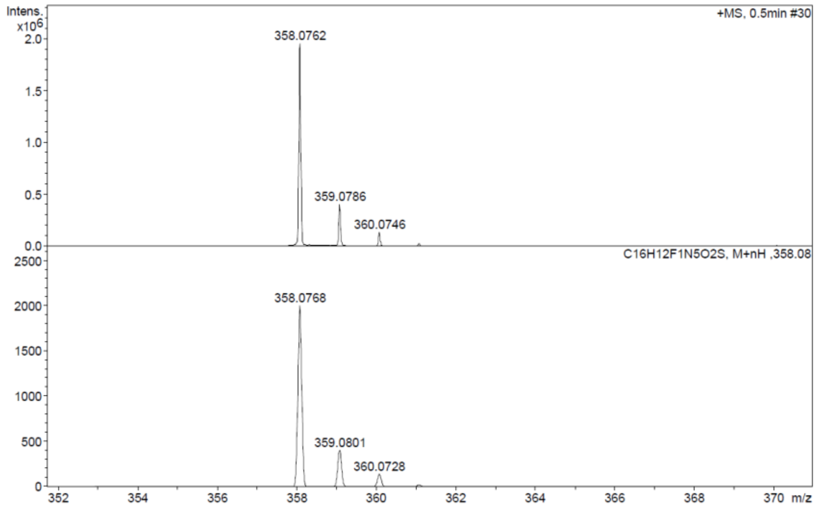
Intens.
x10^6
+MS, 0.5min #30
358.0762
359.0786
360.0746
C16H12F1N5O2S, M+nH ,358.08
358.0768
359.0801
360.0728
m/z

4f

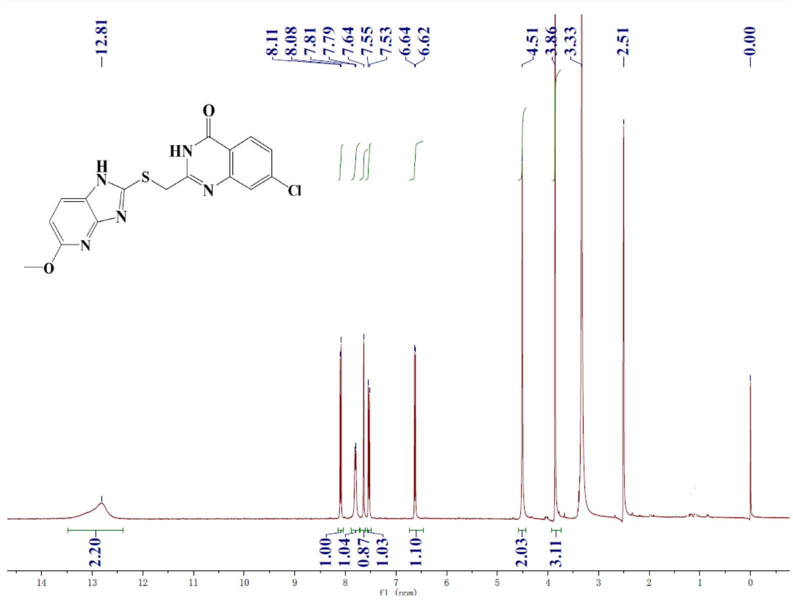
12.81
8.11
8.08
7.81
7.79
7.64
7.55
7.53
6.64
6.62
4.51
3.86
3.33
2.51
0.00
O
HN
H
N
S
N
Cl
N
N
O
2.20
1.00
1.04
0.87
1.03
1.10
2.03
3.11
f1 (ppm)

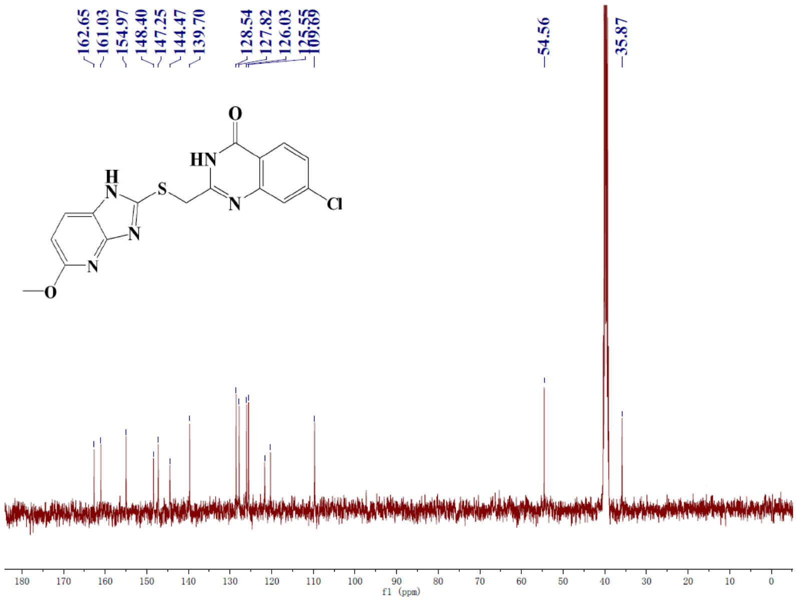
162.65
161.03
154.97
148.40
147.25
144.47
139.70
128.54
127.82
126.03
54.56
35.87
O
HN
H
N
S
N
Cl
N
N
O
f1 (ppm)

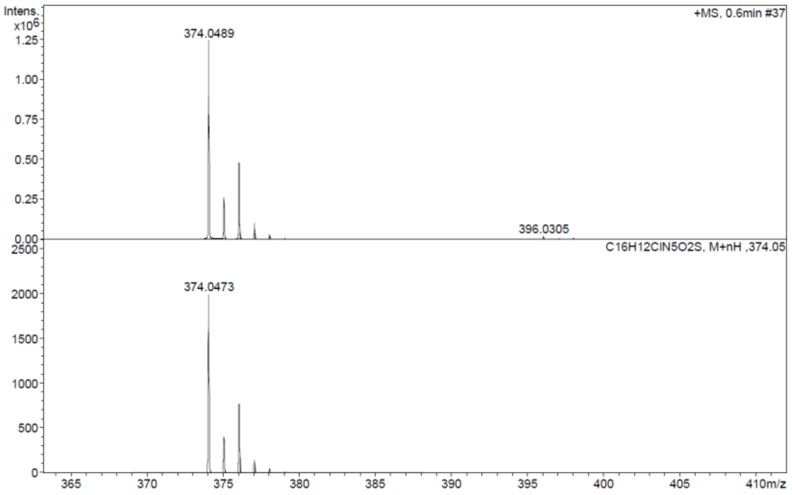
Intens.
x10^6
+MS, 0.6min #37
374.0489
396.0305
C16H12ClN5O2S, M+nH ,374.05
374.0473
m/z

4g

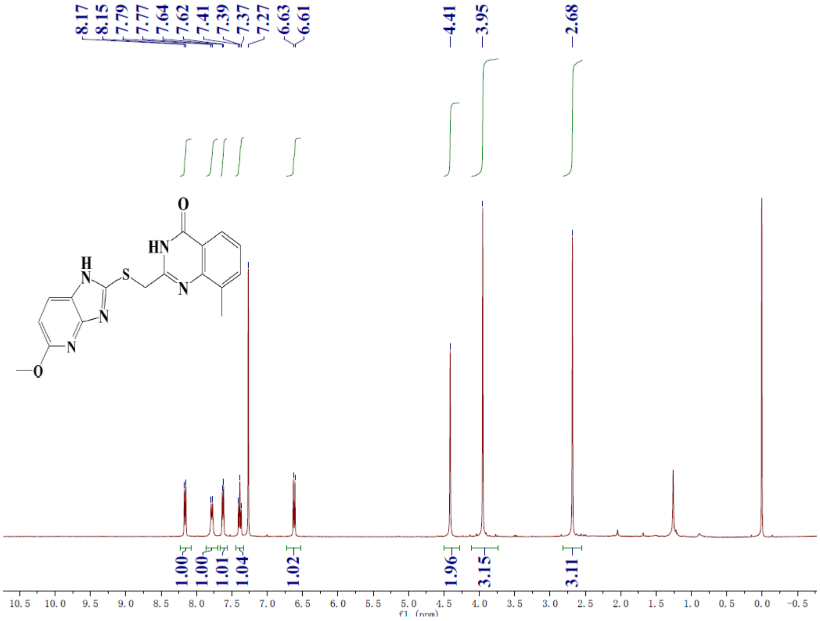

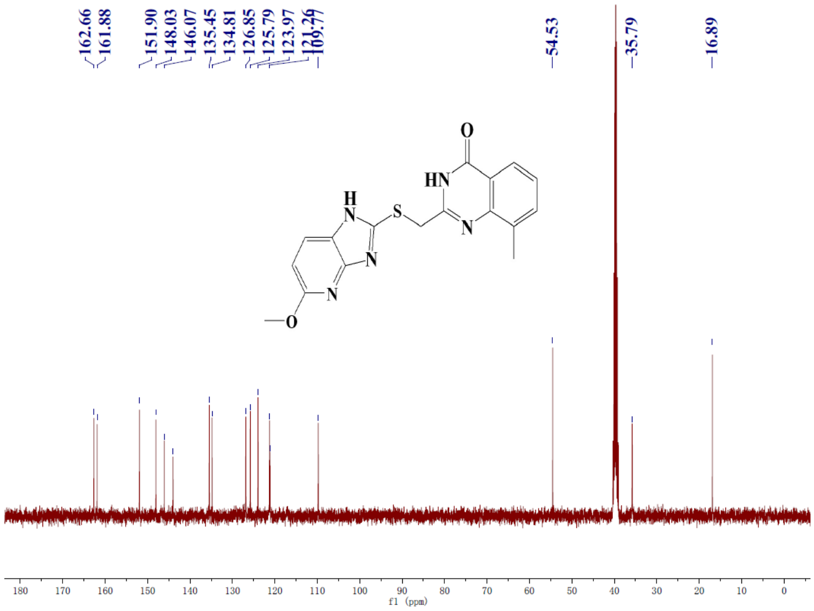

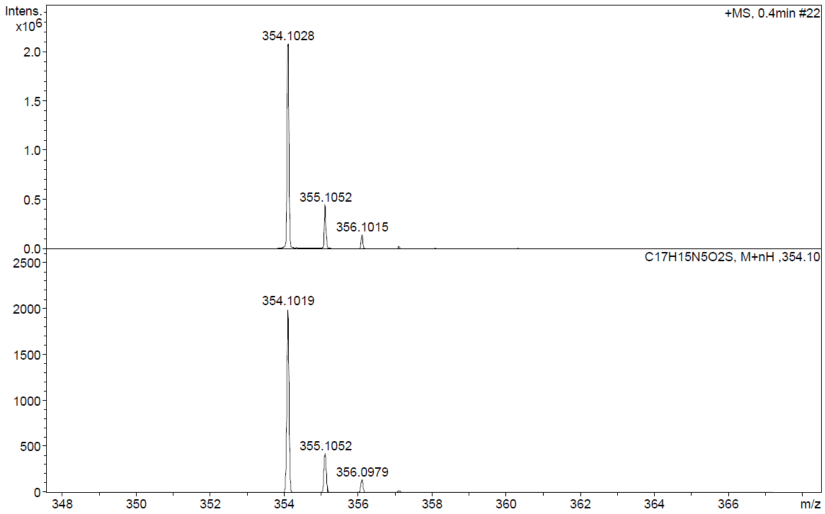
Intens.
x10^6
+MS, 0.4min #22
354.1028
355.1052
356.1015
C17H15N5O2S, M+nH ,354.10
354.1019
355.1052
356.0979
m/z

4h

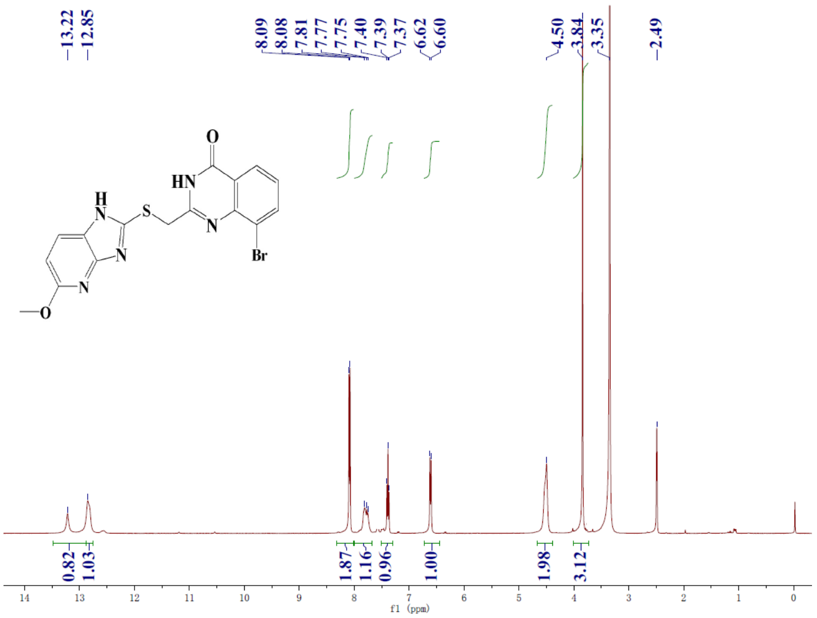
13.22
12.85
8.09
8.08
7.81
7.77
7.75
7.40
7.39
7.37
6.62
6.60
4.50
3.84
3.35
2.49
0.82
1.03
1.87
1.16
0.96
1.00
1.98
3.12
f1 (ppm)

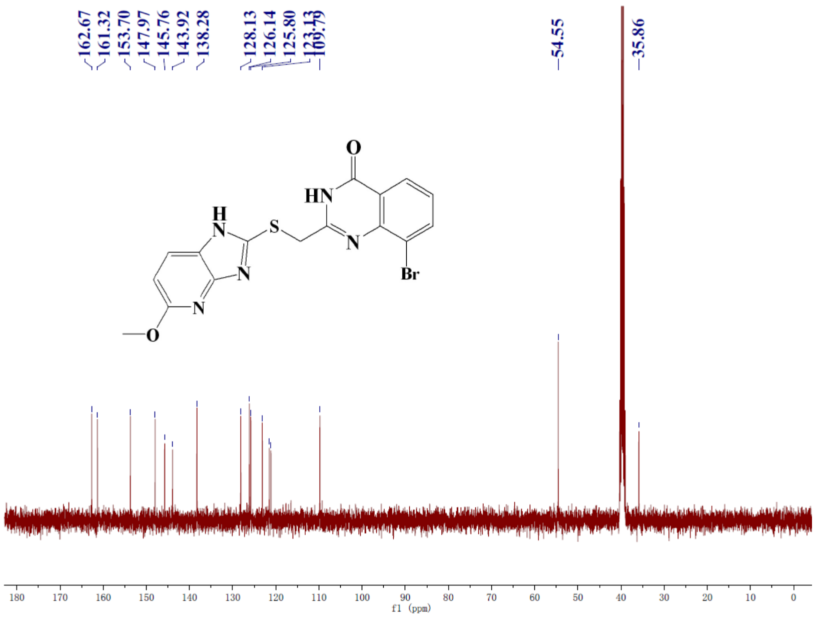
162.67
161.32
153.70
147.97
145.76
143.92
138.28
128.13
126.14
125.80
54.55
35.86
f1 (ppm)

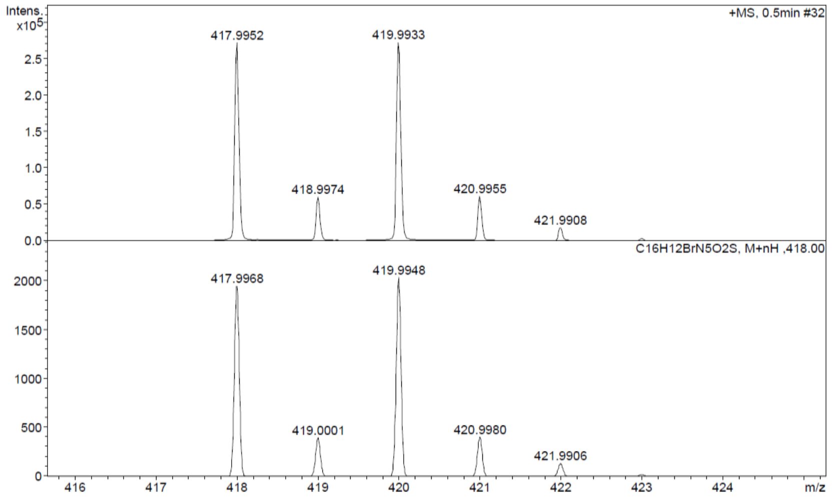
Intens.
x10^5
+MS, 0.5min #32
417.9952
418.9974
419.9933
420.9955
421.9908
C16H12BrN5O2S, M+nH ,418.00
417.9968
419.0001
419.9948
420.9980
421.9906
m/z

Gene sequence: (2582 bp)

GAATTCGCCGCCACCATGGGCGTGCTGCTGACACAGAGGACACTGCTGT
CCCTGGTGCTGGCCCTGCTGTTCCCTAGCATGGCCTCCATGGGCAGGTGTT
TTGAGAGGACATTTGGCAACTGCAGATGTGATGCCGCCTGCGTGGAGCTG
GGCAATTGTTGCCTGGACTACCAGGAGACCTGCATCGAGCCTGAGCACATC
TGGACCTGCAATAAGTTTAGGTGCGGCGAGAAGAGACTGACCAGGAGCCT
GTGTGCCTGTTCCGACGACTGCAAGGATAAGGGCGACTGCTGCATCAACTA
CAGCTCCGTGTGCCAGGGCGAGAAGAGCTGGGTGGAGGAGCCCTGCGAG
TCCATCAATGAGCCCCAGTGCCCTGCCGGCTTCGAGACCCCACCTACACTG
CTGTTTTCCCTGGATGGCTTTAGAGCCGAGTACCTGCACACATGGGGCGGC
CTGCTGCCTGTGATCTCCAAGCTGAAGAAGTGTGGCACCTACACCAAGAA
CATGAGACCCGTGTACCCTACCAAGACATTTCCTAATCACTACTCCATCGTG
ACAGGCCTGTACCCTGAGAGCCACGGCATCATCGATAATAAGATGTACGAT
CCTAAGATGAACGCCTCCTTCTCCCTGAAGTCCAAGGAGAAGTTCAACCCT
GAGTGGTACAAGGGCGAGCCCATCTGGGTGACAGCCAAGTACCAGGGCCT
GAAGTCCGGCACCTTTTTCTGGCCTGGCAGCGACGTGGAGATCAACGGCA
TCTTTCCTGATATCTACAAGATGTACAACGGCAGCGTGCCCTTCGAGGAGA
GGATCTTGGCCGTGCTGCAGTGGCTGCAGCTGCCCAAGGACGAGAGGCCT
CACTTCTACACCCTGTACCTGGAGGAGCCTGACTCCTCCGGCCACAGCTAC
GGCCCCGTGAGCAGCGAGGTCATTAAGGCCCTGCAGAGGGTGGACGGCAT
GGTGGGCATGCTGATGGACGGCCTGAAGGAGCTGAATCTGCACAGATGTC
TGAATCTGATCCTGATCTCCGACCACGGCATGGAGCAGGGCAGCTGCAAG
AAGTACATCTACCTGAACAAGTACCTGGGCGATGTGAAGAATATCAAGGTC
ATCTATGGCCCCGCCGCCAGGCTGAGACCCAGCGACGTTCCCGATAAGTAC
TACTCCTTCAACTACGAGGGCATCGCCAGGAATCTGTCCTGTAGAGAGCCT
AACCAGCACTTTAAGCCCTACCTGAAGCACTTCCTGCCTAAGAGGCTGCAC
TTTGCCAAGAGCGATAGAATCGAGCCTCTGACCTTTTACCTGGACCCCCAG
TGGCAGCTGGCCCTGAATCCTAGCGAGAGGAAGTACTGTGGCTCCGGCTTT
CACGGCTCCGATAACGTGTTCTCCAACATGCAGGCCCTGTTCGTGGGCTAC
GGCCCCGGATTCAAACACGGCATCGAGGCCGATACATTCGAGAATATCGAG
GTGTACAACCTGATGTGTGATCTGCTGAACCTGACCCCCGCCCCTAACAAT
GGCACCCACGGCTCCCTGAACCACCTGCTGAAGAATCCTGTGTACACCCCT
AAGCACCCCAAGGAGGTGCACCCTCTGGTGCAGTGCCCTTTTACAAGAAA
TCCCAGGGATAATCTGGGCTGCAGCTGTAACCCTAGCATCCTGCCTATCGAG

GACTTTCAGACCCAGTTTAATCTGACAGTGGCCGAGGAGAAGATCATCAAG
CACGAGACCCTGCCTTACGGCAGACCTAGGGTGCTGCAGAAGGAGAACAC
CATCTGCCTGCTGTCCCAGCACCAGTTCATGTCCGGCTACTCCCAGGACAT
CCTGATGCCTCTGTGGACAAGCTACACAGTGGACAGAAACGATTCCTTTAG
CACCGAGGACTTTAGCAATTGTCTGTACCAGGATTTTAGAATCCCCCTGAG
CCCCGTGCACAAGTGCTCCTTTTACAAGAATAATACCAAGGTGTCCTACGG
CTTCCTGAGCCCCCCCCAGCTGAACAAGAACTCCTCCGGCATCTACAGCGA
GGCCCTGCTGACAACCAATATCGTGCCTATGTACCAGTCCTTCCAGGTCATT
TGGAGGTACTTCCACGATACCCTGCTGAGGAAGTACGCCGAGGAGAGAAA
TGGCGTGAACGTGGTGAGCGGCCCTGTGTTTGACTTCGATTACGATGGCAG
GTGTGATAGCCTGGAGAACCTGAGACAGAAGAGAAGGGTCATTAGGAACC
AGGAGATCCTGATCCCCACCCACTTCTTTATCGTGCTGACAAGCTGCAAGG
ATACCTCCCAGACACCTCTGCACTGCGAGAACCTGGATACCCTGGCCTTTAT
CCTGCCCCACAGGACCGACAATTCCGAGAGCTGTGTGCACGGCAAGCACG
ACTCCTCCTGGGTGGAGGAACTGCTGATGCTGCACAGAGCCAGAATCACA
GACGTGGAGCACATCACAGGCCTGTCCTTCTACCAGCAGAGGAAGGAGCC
TGTGTCCGATATCCTGAAGCTGAAGACACACCTGCCTACATTCAGCCAGGA
GGACGGCGGCAGCCACCACCACCACCATCACCACCACTAATAGGATCC

Peptide sequence: (852 aa)

MGVLLTQRTLLSLVLALLFPSMASMGRCFERTFGNCRCDAACVELGNCCL
DYQETCIEPEHIWTCNKFRCGEKRLTRSLCACSDDCKDKGDCCINYSSVCQGE
KSWVEEPCESINEPQCPAGFETPPTLLFSLDGFRAEYLHTWGGLLPVISKLKKC
GTYTKNMRPVYPTKTFPNHYSIVTGLYPESHGIIDNKMYDPKMNASFSLKSKE
KFNPEWYKGEPIWVTAKYQGLKSGTFFWPGSDVEINGIFPDIYKMYNGSVPFE
ERILAVLQWLQLPKDERPHFYTLYLEEPDSSGHSYGPVSSEVIKALQRVDGMV
GMLMDGLKELNLHRCLNLILISDHGMEQGSCKKYIYLNKYLGDVKNIKVIYG
PAARLRPSDVPDKYYSFNYEGIARNLSCREPNQHFKPYLKHFLPKRLHFAKSD
RIEPLTFYLDPQWQLALNPSERKYCGSGFHGSDNVFSNMQALFVGYGPGFKH
GIEADTFENIEVYNLMCDLLNLTPAPNNGTHGSLNHLLKNPVYTPKHPKEVHP
LVQCPFTRNPRDNLGCSCNPSILPIEDFQTQFNLTVAEEKIIKHETLPYGRPRVL
QKENTICLLSQHQFMSGYSQDILMPLWTSYTVDRNDSFSTEDFSNCLYQDFRIP
LSPVHKCSFYKNNTKVSYGFLSPPQLNKNSSGIYSEALLTTNIVPMYQSFQVIW
RYFHDTLLRKYAEERNGVNVVSGPVFDFDYDGRCDSLENLRQKRRVIRNQEI
LIPTHFFIVLTSCKDTSQTPLHCENLDTLAFILPHRTDNSESCVHGKHDSSWVE
ELLMLHRARITDVEHITGLSFYQQRKEPVSDILKLKTHLPTFSQEDGGSHHHH
HHHH
